# Asynchronous recovery of water relations and photosynthesis following natural rainfall pulses in *Eucalyptus*

**DOI:** 10.1093/treephys/tpag016

**Published:** 2026-02-02

**Authors:** Edith J Singini, David Drew

**Affiliations:** Department of Forestry and Wood Sciences, Bosman St, Stellenbosch University, Private Bag X1, Matieland 7600, Western Cape, South Africa; Botany Department, 3230 Lucas Ave, Rhodes University, PO Box 94, Grahamstown 6140, Eastern Cape, South Africa; Department of Forestry and Wood Sciences, Bosman St, Stellenbosch University, Private Bag X1, Matieland 7600, Western Cape, South Africa

**Keywords:** anisohydric-isohydric continuum, drought resilience, gas exchange, metabolic limitation, stomatal limitation

## Abstract

Rainfall pulses create brief but critical opportunities for carbon uptake in seasonally dry forests; however, how tree seedlings recover photosynthetic function during the establishment phase following these short-lived rewetting events under field conditions remains poorly understood. In particular, the coordination and timing of hydraulic, stomatal and biochemical recovery processes during natural rainfall pulse–dry-down cycles are not well quantified, despite their importance for carbon-water coupling and drought resilience. Here, we investigated short-term physiological responses of establishing *Eucalyptus* seedlings during naturally occurring rainfall pulse–dry-down cycles. We measured leaf water potential (Ψ_leaf_), gas exchange and photosynthetic capacity (V*_cmax_*, J*_max_*) before and after rainfall to assess recovery dynamics of diffusional and biochemical processes under contrasting atmospheric demand. Across species, Ψ_leaf_ and stomatal conductance improved rapidly following rainfall, reflecting transient hydraulic relief, while net photosynthesis increased by 40–60% within 1–3 days. In contrast, biochemical capacity responded more gradually: V*_cmax_* declined by up to ~ 15% and J*_max_* by 20–40% during dry-down and showed limited or partial recovery after rewetting. Limitation partitioning revealed asynchronous recovery, with stomatal limitation relaxing rapidly after rainfall under low vapour pressure deficit (VPD), whereas under high VPD, biochemical recovery preceded full stomatal reopening. The xeric-origin *Eucalyptus cladocalyx* sustained assimilation at more negative Ψ_leaf_ and exhibited greater biochemical stability, whereas intermediate and mesic species (*E. grandis*, *E. urophylla*, *E. cloeziana*) showed rapid but short-lived post-rain responses. Together, these results demonstrate that photosynthetic recovery during the seedling phase is asynchronous and strongly modulated by atmospheric demand, shaping short-term carbon-water coupling under increasingly pulsed hydroclimates.

## Graphical Abstract

**Graphical Abstract:**
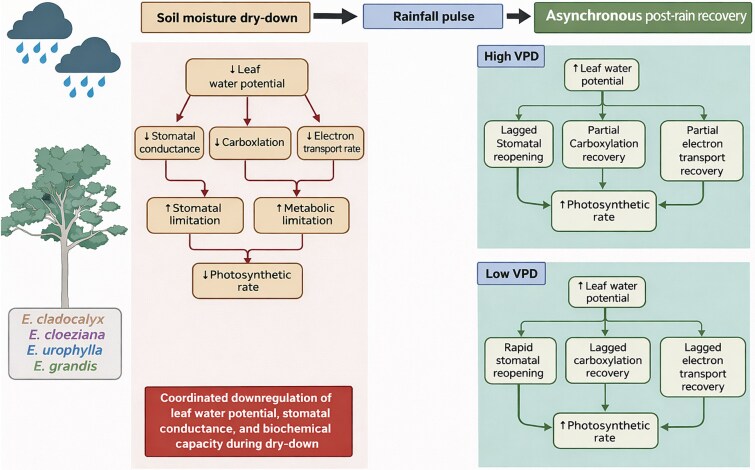

## Introduction

Climate change is intensifying the frequency and magnitude of both droughts and extreme rainfall events, reshaping the temporal distribution of water availability across terrestrial ecosystems (IPCC 2021). In seasonally dry and Mediterranean-type regions, plants increasingly experience water supply as intermittent rainfall pulses separated by prolonged dry periods, rather than as continuous moisture availability (Huxman et al. 2004, Zeppel et al. 2014). These pulse–dry-down dynamics impose rapid alternations between phases of hydraulic relief and renewed water limitation, exerting strong but transient effects on photosynthetic performance and carbon–water exchange (Martínez-Vilalta and Garcia-Forner 2017, Kannenberg et al. 2022). The ability of plants to capitalize on short-lived rewetting events, while maintaining function during subsequent drying, represents a key determinant of drought resilience under intensifying hydroclimatic variability.

Rainfall pulses impose alternating phases of hydraulic relief and carbon limitation, and the sequence and speed of these transitions determine the persistence of drought legacies in photosynthesis (Ruehr et al. 2019, Rehschuh et al. 2020). Following rewetting, hydraulic conductance and leaf water potential often recover more rapidly than photosynthetic metabolism, resulting in a temporary mismatch between water status and carbon assimilation (Skelton et al. 2017). This mismatch reflects differing recovery rates of hydraulic and stomatal processes regulating CO_2_ supply, versus biochemical limitations associated with carboxylation and electron transport. This asynchrony arises because diffusional (stomatal) processes respond almost immediately to changes in water availability, whereas biochemical recovery of carboxylation and electron transport is slower, requiring deactivation and repair of enzymatic machinery damaged during drought (Flexas et al. 2009, Morabito et al. 2022). Understanding how these sequential processes unfold during natural rainfall events is essential for predicting how trees respond to fluctuating moisture inputs in situ. Controlled drought and rewatering experiments, though fundamental for elucidating physiological mechanisms (Héroult et al. 2013, Martorell et al. 2014), often impose uniform or stepwise changes in soil moisture under constrained atmospheric conditions, which may not capture the coupled soil–atmosphere dynamics characteristic of natural rainfall pulse–dry-down cycles (Singini et al. 2026). As a result, such approaches can overlook how interactions between soil moisture and atmospheric demand shape recovery in the field (Singini et al. 2026). Quantifying the recovery of leaf water status, stomatal regulation and photosynthetic capacity under natural rainfall events provides an opportunity to capture ecologically realistic recovery kinetics that emerge from the joint dynamics of hydraulic, stomatal and biochemical processes—linkages that current ecosystem and land-surface models still fail to represent mechanistically (Grossiord et al. 2020, Kannenberg et al. 2022).

Species differ widely in their capacity to regulate water loss and sustain photosynthesis across wetting–drying cycles. Along the isohydric–anisohydric continuum (Martínez-Vilalta et al. 2014, McCulloh et al. 2019), isohydric species maintain relatively stable leaf water potential by closing stomata early during water deficit, thereby reducing hydraulic risk but limiting carbon gain. In contrast, anisohydric species allow leaf water potential to decline, maintaining photosynthesis at lower water potentials while incurring greater hydraulic strain (Brodribb et al. 2010, Meinzer et al. 2017). These contrasting regulatory strategies represent trade-offs between hydraulic safety and carbon productivity, which become especially evident during short-term rainfall pulses when both soil and atmospheric moisture conditions shift rapidly.

Few studies have linked field-based rewetting responses to established physiological frameworks of isohydric–anisohydric regulation (Martínez-Vilalta and Garcia-Forner 2017, Meinzer et al. 2017, Skelton et al. 2017, Ruehr et al. 2019). Rainfall pulses are ecologically significant because they often rehydrate tissues without fully replenishing soil water stores, creating transient phases of carbon assimilation that exert a relatively large influence on seasonal productivity (Huxman et al. 2004, Zeppel et al. 2014). In trees, the speed and completeness of recovery determine whether photosynthetic carbon uptake and growth recover or remain suppressed, a dynamic central to the concept of drought legacy effects (Anderegg et al. 2016, Ruehr et al. 2019). Yet most experimental studies rely on controlled rewatering regimes that fail to capture the coupled variability of soil and atmospheric drivers (Grossiord et al. 2020, Singini et al. 2026). Quantifying physiological responses under field-realistic pulse–dry-down cycles therefore provides a more accurate mechanistic basis for predicting tree resilience and carbon–water coupling in seasonally dry environments.

The genus *Eucalyptus* provides an ideal model for exploring how climatic origin shapes these physiological adjustments. Species from xeric regions typically display conservative water-use traits and high tolerance to low leaf water potential, while mesic-origin species exhibit greater plasticity but reduced drought tolerance (Héroult et al. 2013, Bourne et al. 2017, Smith et al. 2023). Although *Eucalyptus* species have been extensively studied under controlled drought and rewatering conditions (Héroult et al. 2013, Bourne et al. 2017, Singini et al. 2026), the coordination of hydraulic recovery, stomatal regulation and biochemical reactivation during naturally occurring rainfall pulse–dry-down cycles remains poorly resolved. While earlier studies on *Eucalyptus* drought responses have provided key insights into recovery mechanisms (Héroult et al. 2013, Martorell et al. 2014, Bourne et al. 2017), these studies were largely conducted under experimental designs that did not explicitly resolve recovery dynamics during naturally occurring rainfall pulse–dry cycles, where soil moisture and atmospheric demand covary. Here, we extend this framework by examining recovery dynamics during natural rainfall pulses, allowing us to assess how hydraulic, stomatal and biochemical processes are coordinated under realistic hydroclimatic variability.

Here, we investigated the short-term physiological responses of four *Eucalyptus* species differing in climatic origin: the xeric *Eucalyptus cladocalyx*, the intermediate *E. cloeziana* and the mesic *E. grandis* and *E. urophylla*, during two naturally occurring rainfall pulse–dry-down cycles under field conditions. We assessed leaf water potential, gas exchange and photosynthetic capacity (V*_cmax_*, J*_max_*) before and after rainfall to disentangle diffusional and biochemical adjustments. To address how photosynthetic function recovers following natural rainfall pulses under realistic hydroclimatic conditions, we posed the following research questions:

(i) Do *Eucalyptus* species differing in climatic origin operate over distinct physiological ranges during soil drying, particularly with respect to gas exchange at declining leaf water potentials?

(ii) How do rainfall pulses influence the relative contributions of diffusional and biochemical limitations to photosynthesis during short-term recovery and subsequent dry-down?

(iii) Does recovery of leaf water status, stomatal conductance and photosynthetic capacity occur synchronously following rainfall, or do these processes recover asynchronously in a manner dependent on species identity and atmospheric demand?

By addressing these questions, this study provides field-based insights into the coordination of water and carbon processes during rainfall pulse–dry-down cycles, a key uncertainty in predicting forest carbon–water dynamics under increasingly variable precipitation regimes.

## Materials and methods

### Study site and species selection

The study was conducted at the EucXylo research plantation, Stellenbosch University, South Africa (33°55′S, 18°51′E; Singini et al. 2025), which has a Mediterranean-type climate characterized by cool, wet winters and hot, dry summers interspersed with episodic, pulsed rainfall events. Mean annual rainfall is ~ 760 mm, concentrated between May and September, and mean daily maximum temperatures range from 16 °C in winter to 30 °C in summer (Lakhraj-Govender and Grab 2019). Soils are classified as Lithic Leptosols (Hengl et al. 2015), characterized by shallow profiles, low water-holding capacity and rapid drainage. Textures vary from loamy fine sand to fine sandy loam, with loamy fine sand dominating across the site (Singini et al. 2025). The combination of shallow, well-drained soils and pulsed summer rainfall generates rapid wetting–drying cycles, providing an ideal framework for assessing short-term physiological responses of trees to episodic water availability under field conditions. Within this context, four *Eucalyptus* seedlings differing in climatic origin and drought strategy were studied: the xeric *E. cladocalyx*, the mesic *E. grandis* and *E. urophylla*, and the intermediate *E. cloeziana* (Table 1). All individuals were ~2 years old at the time of measurements.

**Table 1:** Hydroclimatic origin and rainfall regimes of the four *Eucalyptus* species studied. Species are ordered along a gradient of native mean annual precipitation (MAP) and rainfall frequency, providing context for interpreting interspecific differences in physiological responses to rainfall pulse–dry-down cycles observed under field conditions

Species	Native region	Native hydroclimate	MAP range (mm)	Rainfall regime	Relevance to pulse study
*E. cladocalyx*	Flinders Ranges, South Australia	Semi-arid Mediterranean	~250–600	Low, episodic	Native to environments characterized by infrequent, episodic rainfall, providing a hydroclimatic context dominated by discrete wetting–drying cycles
*E. cloeziana*	Queensland forests	Subtropical mesic	~900–1400	Seasonal	Originates from seasonally mesic environments with periodic dry spells, offering an intermediate context between episodic and frequently wetted systems
*E. grandis*	Eastern coastal Australia	Mesic-wet	~1200–2000	Frequent	Native to mesic–wet regions with relatively frequent rainfall, representing limited exposure to extended dry-down between rainfall events
*E. urophylla*	Timor and Indonesia	Tropical mesic	~1500–2500	Frequent	Originates from high-rainfall tropical regions with frequent moisture inputs, representing minimal exposure to pulse-driven water availability

### Experimental design and sampling framework

All species were planted at 5 × 5 m spacing (400 trees ha^−1^) within a common experimental block, with each species occupying a discrete plot measuring 30 × 45 m. Each species plot contained 70 trees, of which 18 centrally located individuals were designated as research trees, and the remaining trees served as buffers to minimize edge effects. From the research trees, five healthy, canopy-exposed individuals per species were selected and repeatedly measured through two naturally occurring rainfall pulse–dry-down cycles: one in early summer (December 2024) and one in late summer (March 2025). Each cycle consisted of a pre-rain dry-down phase (−37 to 0 and −36 to 0 days before rainfall) followed by a post-rain recovery and subsequent drying phase (1, 3, 5 days after and 1, 4, 18 days after rainfall, respectively; Table 2). The December pulse occurred under relatively low vapour pressure deficit (VPD) conditions, whereas the March event coincided with higher atmospheric demand. Although only two rainfall pulse–dry-down cycles were captured during the monitoring period, they occurred under contrasting evaporative conditions, providing a natural experiment to assess how atmospheric demand modulates short-term physiological recovery following rainfall.

**Table 2:** Summary of sampling periods and environmental phases monitored during two naturally occurring rainfall events in December 2024 and March 2025

Period	Phase	Sampling days (relative to rainfall)	Conditions captured
December (2024)	Dry-down	–37 to 0 days before rainfall	Early summer end of the drying period
	Post-pulse	1, 3, 5 days after rain	Recovery under low VPD
March (2025)	Dry-down	−36 to 0 days before rainfall	Late-summer end of the drying period
	Post-pulse	1, 4, 18 days after rain	Recovery under high VPD

### Leaf water potential and gas exchange measurements

Predawn and midday leaf water potential (Ψ_leaf_) were measured on each sampling day using a Scholander-type pressure chamber (Model 1505D, PMS Instrument Co., Albany, OR, USA). Predawn measurements were conducted between 03:00 and 05:00 h, while midday measurements were taken between 11:00 and 13:00 h. Leaves were immediately excised from sun-exposed upper-canopy positions, immediately sealed in plastic bags containing moist paper towels, and measured within 1 h of excision. Predawn values were interpreted as indicators of soil–plant hydraulic equilibrium, whereas midday values reflected the combined effects of soil water availability and atmospheric demand.

Leaf gas exchange and *A*C**_i_ (net CO_2_ assimilation rate, A, versus intercellular CO_2_ concentration, C_i_) curves were measured between 09:00 and 15:00 h on clear days using a portable photosynthesis system (LI-6800; LI-COR Biosciences, Lincoln, NE, USA). Measurements were conducted on fully expanded, sunlit leaves from the upper canopy to ensure consistent light acclimation and minimize microclimatic variability. After stabilization in the chamber, AC_i_ curves were obtained using a dynamic CO_2_ ramping protocol, in which the reference CO_2_ was continuously increased from 50 to 1800 p.p.m. Data were logged at 1-min intervals, yielding an average of 103 observations per curve over ~ 7 min. This approach allowed all five individuals per species to be measured within a single day. The order of species, plots and individuals was randomized to minimize diurnal bias. Chamber conditions were maintained close to ambient field conditions: reference CO_2_ (C_a_) = 420 μmol conditions, photosynthetic photon flux density = 2000 μmol m^−2^ s^−1^ and leaf temperature = 30 °C. Chamber VPD was allowed to track prevailing atmospheric conditions (typically 1.3–1.8 kPa) within ±0.2 kPa to maintain leaf energy balance. Variation in assimilation (*A*) and stomatal conductance (g_sw_), therefore, reflected plant physiological status rather than chamber artefacts.

### Environmental measurements

Volumetric soil water content (SWC, %) was recorded using Sentek Drill-and-Drop sensors (Sentek Technologies, Stepney, South Australia) installed at 60 cm depth in the centre of each species plot. This depth integrates soil moisture variation through the main rooting zone of young *Eucalyptus* trees and captures the progression of wetting and drying after rainfall events. Although surface soils (<20 cm) dry more rapidly, preliminary readings confirmed that temporal trends at 60 cm tracked overall profile dynamics. Surface soils were also monitored periodically and showed similar temporal patterns. Sensors were connected to CR300 data loggers (Campbell Scientific Inc., Logan, UT, USA), programmed to record data every 15 min.

Meteorological data were obtained from an automatic weather station (HOBO U30 NRC, Onset Computer Corp., Bourne, MA, USA) located less than 200 m from the experimental plots. The station was equipped with a tipping-bucket rain gauge (HOBO S-RG3-M), an air-temperature and relative-humidity probe (HOBO S-THB-M002) and a silicon-cell pyranometer (HOBO S-LIA-M003) for incident solar radiation. Data were logged at 15-min intervals. The VPD (kPa) was calculated from these temperature and humidity records following Buck (1981) and averaged over the measurement window corresponding to each physiological sampling day to characterize ambient atmospheric demand.

### Estimation of photosynthetic parameters and limitation partitioning

AC_i_ curves were fitted to the Farquhar–von Caemmerer–Berry model (FvCB; Farquhar et al. 1980) using the ‘*msuRACiFit*’ package in R, version 4.4.2 (Gregory et al. 2021, R Core Team 2024). Mesophyll conductance (g_m_) was assumed to be constant across treatments (days before or after rainfall) based on previous work showing limited variability in g_m_ under moderate drought for these species (Héroult et al. 2013). Triose-phosphate-utilization (TPU) limitation detection was activated in the fitting routine; curves showing clear TPU limitation at high C_i_ were excluded from V*_cmax_* and J*_max_* estimation, since the presence of a TPU plateau violates the Rubisco- and electron-transport-limited assumptions of the FvCB model and can bias parameter estimates (Sharkey et al. 2007, Gregory et al. 2021). Model parameters were temperature-corrected to account for leaf temperature, as described by Bernacchi et al. (2001). All rates are expressed in μmol m^−2^ s^−1^. Percentage changes in photosynthetic parameters (*A*, g_sw_, V*_cmax_*, J*_max_*) were calculated relative to the pre-rain (dry-down endpoint) values within each species, using the formula ((post-rain − pre-rain)/pre-rain) × 100, to quantify recovery and subsequent declines during drying.

To link changes in photosynthetic capacity with underlying regulatory mechanisms, the relative contributions of stomatal (R_sl_) and metabolic (R_ml_) limitations to photosynthesis were assessed following the baseline-scaled framework of Ripley et al. (2007). Partitioning of stomatal versus non-stomatal controls on photosynthesis under drought has a long conceptual basis (Farquhar and Sharkey 1982, Grassi and Magnani 2005, Flexas et al. 2009), and baseline-scaled indices such as those proposed by Ripley et al. (2007) have been used as descriptive tools to contextualize recovery dynamics alongside direct estimates of biochemical capacity.

For each fitted AC_i_ curve, the modelled net assimilation rate was extracted at the reference atmospheric CO_2_ concentration (C_a_ = 420 p.p.m.) and at the corresponding intercellular CO_2_ concentration (C_i_). Following the notation of Ripley et al. (2007), assimilation at C_a_ under high water availability (defined as the species-specific mean measured after rainfall) was denoted as X, assimilation at C_i_ under the focal condition (dry-down or recovery) as Y, and assimilation at C_a_ under the focal condition as Z. These quantities were derived consistently from the fitted FvCB model.

Stomatal limitation (sl, %) was calculated at the individual-curve level as:


$$ sl=\frac{A_{Ca}-{A}_{Ci}}{A_{Ca}}\times 100. $$

Relative stomatal limitation (R_sl_) was calculated as:


$${R}_{sl}=\frac{Y-Z}{X}\times100. $$

Relative metabolic limitation (R_ml_) was calculated as:


$$ {R}_{ml}=\frac{X-Z}{X}-{R}_{sl}\times 100 $$

We note that R_sl_ and R_ml_ represent baseline-scaled indices rather than strictly mechanistic partitioning of photosynthetic limitation. Accordingly, the interpretation of biochemical limitation and recovery dynamics in this study is based primarily on direct estimates of photosynthetic capacity (V*_cmax_* and J*_max_*), with R_ml_ used only as a complementary descriptor of baseline-scaled non-stomatal constraint.

### Data analysis

Analyses of Ψ_leaf_, *A*, g_sw_, photosynthetic parameters (V*_cmax_* and J*_max_*) and photosynthetic limitations (R_sl_ and R_ml_) were conducted across species and measurement days corresponding to periods before and after rainfall events. All statistical analyses were performed in R. Before analysis, all response variables were visually inspected for normality and homogeneity of variance using quantile–quantile (Q–Q) plots and residual-versus-fitted value diagnostics. Where necessary, variables such as g_sw_, *A* and the relative limitation indices (R_sl_, R_ml_) were log-transformed to improve model fit and better meet assumptions of homoscedasticity. Mixed-effects models were fitted separately for each month, with species and measurement day as fixed effects, and plant ID (nested within plot) included as a random factor to account for repeated measurements on the same individuals. For leaf water potential, models included species, measurement day and time of day (predawn or midday) as fixed effects, with plant ID (nested within plot) again treated as a random factor. This approach enabled testing for species- and time-dependent responses without directly comparing across months, as measurement days differed between the December and March cycles. Type III Wald χ^2^ tests were used to assess the significance of main effects and interactions (‘car’ package; Fox and Weisberg 2018). When significant effects were detected, post hoc pairwise comparisons were conducted using the ‘emmeans’ package (Lenth et al. 2018) with Tukey’s HSD adjustment for multiple comparisons. Targeted treatment-versus-control contrasts compared post-rainfall days with the dry-down baseline within each species, applying Holm’s correction for multiple testing. Model residuals were examined with the ‘DHARMa’ package (Hartig 2016) to verify normality, homoscedasticity and model fit. All estimated marginal means were back-transformed to the original scale using bias-adjusted inverse links, and significance was accepted at α = 0.05. Full model outputs, including Type III ANOVA tables and post hoc contrasts, are provided in Tables S1–S5 available as Supplementary Data at *Tree Physiology* Online.

## Results

### Rainfall pulse events and soil-moisture dynamics

**Figure 1:**
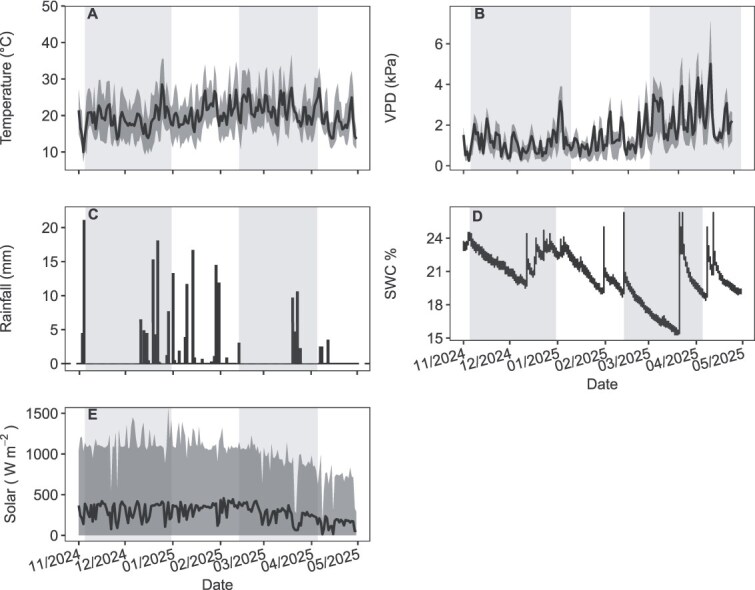
Environmental conditions recorded at the EucXylo research plantation (Stellenbosch, South Africa) from November 2024 to April 2025. Grey-shaded regions denote the two monitored rainfall pulse–dry-down events (December and March), during which physiological measurements were conducted at pre-rain (baseline) and at +1, +3, +5 and + 18 days following rainfall, depending on the event. Time series illustrate temporal variation in key climatic drivers and soil moisture, capturing periods of progressive drying and subsequent rewetting. (A) Air temperature, with the daily minimum–maximum range shown as a shaded band and the daily mean shown as a solid line. (B) Daytime vapour pressure deficit (VPD; 07:00–17:00 h), with the daily minimum–maximum range shown as a shaded band and the daily mean shown as a solid line. (C) Daily rainfall totals (midnight–midnight). (D) Volumetric soil water content (SWC, %), measured in open inter-row areas and representing site-level soil moisture availability rather than conditions directly beneath tree canopies. (E) Incoming solar radiation (W m^−2^), with the daily minimum–maximum range shown as a shaded band and the daily mean shown as a solid line. Trace rainfall events (< 1 mm) are displayed but were excluded from calculations used to define dry-down periods.

Environmental conditions between November 2024 and April 2025 were characterized by two comparable wetting–drying cycles, one in early to mid-December 2024 and another in mid-February to late March 2025, each spanning ~5 weeks (Figure 1). Although both periods were similar in duration, they differed in intensity and atmospheric demand. Air temperature followed a typical seasonal trajectory, averaging 19.3 °C during the first measurement period (8.9–34.8 °C) and increasing to 22.6 °C during the second period (10.7–36.9 °C; Figure 1A). Rainfall occurred in discrete pulses (Figure 1C), totalling 46.8 mm in December (maximum daily 19.5 mm) and 33.1 mm in February–March (maximum daily 10.2 mm).

The SWC closely tracked these rainfall inputs (Figure 1D), increasing rapidly after rainfall and declining gradually during subsequent dry-downs. During the December cycle, SWC ranged from 19.8% to 24.6% (mean = 22.4%), whereas during February–March, SWC varied between 15% and 23.7% (mean = 19.2%), reflecting lower soil moisture levels later in the season (Figure 1D). Mean daytime VPD averaged 1.15 kPa in December and increased to 2.48 kPa in March, with daily maxima reaching 3.91 kPa and 7.12 kPa, respectively, indicating markedly higher atmospheric demand during late summer (Figure 1B). Solar radiation remained high throughout the monitoring period (Figure 1E). Mean daytime irradiance (09:00–15:00 h) across the sampling days within each campaign, was 339 W m^−2^ in December and 301 W m^−2^ in February–March, with corresponding daily maxima of 1178 W m^−2^ and 1003 W m^−2^, respectively.

### Plant water status during dry-down and rainfall-pulse events

**Figure 2:**
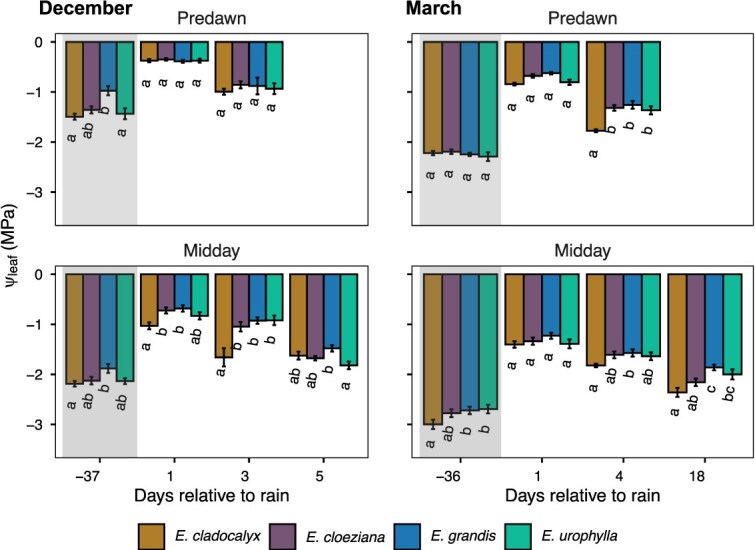
Predawn and midday leaf water potential (Ψ_leaf_) of four *Eucalyptus* species measured at multiple time points before (shaded bars) and after (unshaded bars) rainfall events in December and March. Measurements correspond to 1, 3 and 5 days after rainfall and 37 days before rainfall in December, and to 1, 4 and 18 days after rainfall and 36 days before rainfall in March (*n* = 5). Error bars indicate the standard error of the mean. Different letters denote statistically significant differences among species within each sampling day (Tukey’s HSD, *P* < 0.05).

Changes in Ψ_leaf_ reflected species-specific responses to soil dryness (before rainfall pulses) and to subsequent rewetting across the two measurement periods (Figure 2). At the end of the dry-down period (36−37 days since the previous rainfall), Ψ_leaf_ was more negative across species, indicating water limitation (Figure 2). Following rainfall, Ψ_leaf_ became less negative at the first post-rain sampling point, reflecting short-term recovery in plant water status, before declining again at later post-rain sampling points as soils re-dried (Figures 1 and 2). Mixed-effects modelling confirmed strong main effects of species (χ^2^ = 50.08, df = 3, *P* < 0.001), time of day (predawn vs midday; χ^2^ = 201.21, df = 1, *P* < 0.001) and month (December vs March; χ^2^ = 253.87, df = 1, *P* < 0.001) on Ψ_leaf_, with no significant interactions (*P* > 0.25; Table S1 available as Supplementary Data at *Tree Physiology* Online). This indicates that the relative responses of species were consistent across months and sampling times. Although the December and March dry-downs were of similar duration (36–37 days), March was characterized by lower soil moisture (minimum ≈ 16%) and higher evaporative demand (Figure 1), and Ψ_leaf_ values were correspondingly more negative across all species, reflecting greater overall water limitation during late summer (Figure 2).

In December, Ψ_leaf_ was more negative at the end of the dry-down period, became less negative at the first post-rain sampling point, and declined again at subsequent post-rain days (Figure 2). At the end of the dry-down period, species differences were evident at predawn, with *E. grandis* exhibiting less negative Ψ_leaf_ than *E. cladocalyx* and *E. urophylla*, while not differing significantly from *E. cloeziana* (Tukey HSD, *P* < 0.05; Figure 2; Table S1 available as Supplementary Data at *Tree Physiology* Online). During the early recovery period (1 and 3 days after rainfall), predawn Ψ_leaf_ did not differ among species (Tukey HSD, *P* > 0.05). At midday, species differences emerged earlier than at predawn. One day after rainfall, *E. cladocalyx* exhibited significantly more negative midday Ψ_leaf_ than *E. cloeziana* and *E. grandis*, while *E. urophylla* showed intermediate values (Tukey HSD, *P* < 0.05; Figure 2; Table S1 available as Supplementary Data at *Tree Physiology* Online). Three days after rainfall, *E. cladocalyx* maintained significantly less negative midday Ψ_leaf_ than the other species, whereas by the later post-rain sampling point (5 days after rainfall), *E. grandis* exhibited the least negative midday Ψ_leaf_, differing significantly from *E. urophylla* (Tukey HSD, *P* < 0.05) but not from *E. cloeziana* and *E. cladocalyx* (Tukey HSD, *P* > 0.05; Figure 2; Table S1 available as Supplementary Data at *Tree Physiology* Online).

In March, Ψ_leaf_ values were consistently more negative than in December (predawn ≈ −1.2 to −1.6 MPa; midday ≈ −2.5 to −3.0 MPa; Figure 2), coinciding with lower soil moisture and higher evaporative demand during late summer. Species divergence occurred earlier and was more pronounced than in December. At predawn, species did not differ immediately following rainfall, but by 4 days after rainfall, *E. cladocalyx* exhibited more negative Ψ_leaf_ than *E. cloeziana*, *E. urophylla* and *E. grandis* (Tukey HSD, *P* < 0.05; Figure 2; Table S1 available as Supplementary Data at *Tree Physiology* Online). At midday, species differences were evident across multiple post-rain sampling points. *Eucalyptus cladocalyx* consistently exhibited the most negative midday Ψ_leaf_, differing significantly from *E. grandis* and *E. urophylla* at several post-rain days, while *E. cloeziana* again showed intermediate values (Figure 2; Table S1 available as Supplementary Data at *Tree Physiology* Online). Across both sampling periods, rainfall resulted in short-lived improvements in leaf water status followed by renewed declines as soils dried, with species-specific differences in the magnitude and timing of these responses.

### Gas exchange and photosynthetic parameters

**Figure 3:**
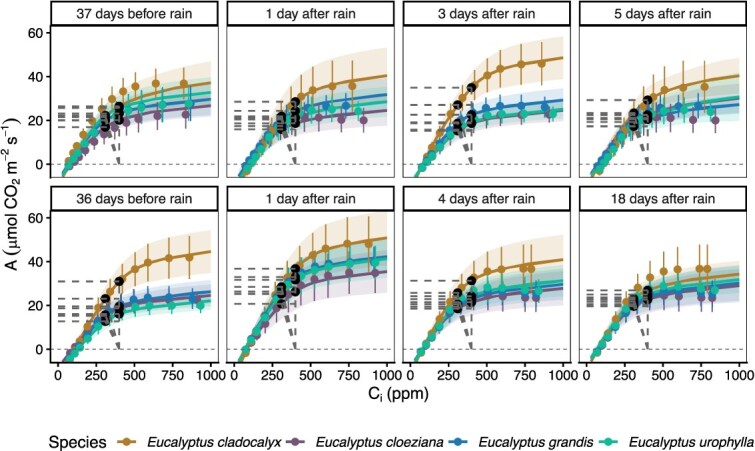
Photosynthetic (*A*) responses to intercellular CO_2_ concentration (C_i_) for four *Eucalyptus* species measured before and after natural rainfall events. AC_i_ curves were measured using a LI-6800 system and fitted with the Farquhar–von Caemmerer–Berry model (Farquhar et al. 1980). The top panel row shows measurements taken 37 days before rainfall and 1, 4 and 5 days after a rainfall during the December dry-down cycle, while the bottom row shows measurements taken 36 days before rainfall and 1, 3 and 18 days after rainfall during the March dry-down cycle. Coloured lines represent species-specific fitted responses, with points showing mean observed values (± SD, *n* = 5). Dashed lines indicate relative stomatal limitation, calculated as the difference between assimilation at ambient CO_2_ concentration (C_a_ = 420 p.p.m.) and assimilation at the corresponding C_i_ for each curve. Together, the panels illustrate species-specific photosynthetic acclimation during prolonged dry-down and subsequent recovery following rainfall under contrasting atmospheric demand.

**Figure 4:**
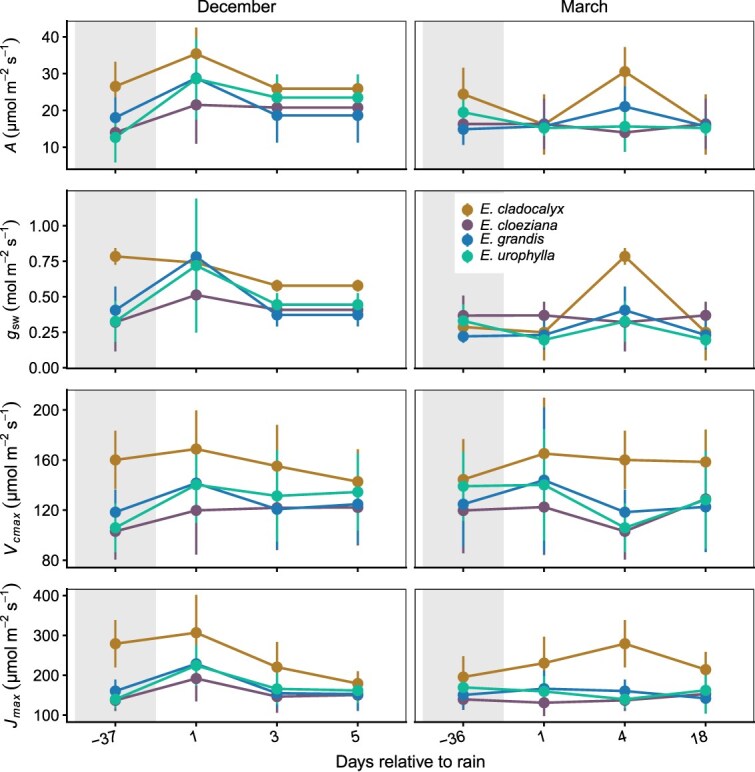
Changes in photosynthetic rate (*A*), stomatal conductance (g_sw_), maximum rate of Rubisco carboxylation (V*_cmax_*) and maximum electron transport rate (J*_max_*) for four *Eucalyptus* species measured before and after rainfall events in December and March. Measurements correspond to 1, 3 and 5 days after rainfall, and 37 days before rain in December, and to 1, 4 and 18 days after rainfall, and 36 days before rain in March (*n* = 5). Shaded regions represent pre-rain (dry-down) conditions, while unshaded regions indicate post-rain recovery. Error bars represent the standard error of the mean.

Following changes in Ψ_leaf_, photosynthetic activity responded to rainfall pulses primarily through temporal variation, with limited and parameter-specific interspecific differences in gas exchange and photosynthetic capacity (Figures 3 and 4). In December, *A* varied significantly among species (χ^2^ = 10.67, df = 3, *P* = 0.0136) and across measurement days (χ^2^ = 26.52, df = 3, *P* < 0.001), with no significant interaction (χ^2^ = 12.28, df = 9, *P* = 0.198; Figure 4; Table S2 available as Supplementary Data at *Tree Physiology* Online). This indicates that *A* differed among species and changed over time; however, species followed similar temporal patterns across the end of the dry-down and rainfall pulse (Figure 4). g_sw_ also showed significant effects of species (χ^2^ = 12.45, df = 3, *P* = 0.0060) and day (χ^2^ = 19.01, df = 3, *P* < 0.001), with a significant interaction (χ^2^ = 24.34, df = 9, *P* = 0.0038), indicating that temporal changes in g_sw_ differed among species (Figure 4). For V*_cmax_*, species had a significant effect (χ^2^ = 9.52, df = 3, *P* = 0.0232), day was marginal (χ^2^ = 7.32, df = 3, *P* = 0.0623) and no significant interaction was detected (χ^2^ = 13.59, df = 9, *P* = 0.137; Table S2 available as Supplementary Data at *Tree Physiology* Online). On the other hand, J*_max_* varied significantly with species (χ^2^ = 20.98, df = 3, *P* < 0.001) and day (χ^2^ = 36.81, df = 3, *P* < 0.001), with a significant interaction (χ^2^ = 21.55, df = 9, *P* = 0.010).

Relative to the end of the dry-down, *A* increased significantly 1 day after rainfall in *E. grandis* (t = 2.75, *P* = 0.025) and in *E. urophylla* at 1, 3 and 5 days after rainfall (t = 3.41, *P* = 0.0042; t = 3.29, *P* = 0.0056; t = 3.29, *P* = 0.0056; respectively; Figure 4; Table S3 available as Supplementary Data at *Tree Physiology* Online). In contrast, g_sw_ increased significantly only in *E. grandis* 1 day after rainfall (t = 3.63, *P* = 0.003). For V*_cmax_*, no significant post-rain contrasts were detected within species. J*_max_* increased significantly 1 day after rainfall in *E. cloeziana* (t = 3.08, *P* = 0.011) and *E. urophylla* (t = 2.56, *P* = 0.043), but decreased by 5 days after rainfall in *E. cladocalyx* (t = 3.67, *P* = 0.003; Table S3 available as Supplementary Data at *Tree Physiology* Online). Species comparisons within each day showed additional differences: at 37 days before rainfall, *E. cladocalyx* had higher g_sw_ than *E. cloeziana* (t = 4.30, *P* < 0.001) and higher *A* than *E. cloeziana* and *E. urophylla* (t = 3.31, *P* = 0.009; t = 3.50, *P* = 0.006). For J*_max_*, *E. cladocalyx* was higher than *E. grandis* and *E. urophylla* (t = 3.40, *P* = 0.007; t = 4.08, *P* = 0.001), and for V*_cmax_*, it was higher than *E. cloeziana* at both 37 days before (t = 3.69, *P* = 0.003) and 1 day after rainfall (t = 2.97, *P* = 0.023; Figure 4).

In March, *A* varied significantly across days (χ^2^ = 408.56, df = 3, *P* < 0.001) but not among species (χ^2^ = 3.19, df = 3, *P* = 0.363), with no species × day interaction (χ^2^ = 1.99, df = 9, *P* = 0.983; Table S2 available as Supplementary Data at *Tree Physiology* Online). g_sw_ also differed significantly across days (χ^2^ = 53.16, df = 3, *P* < 0.001), while species and the interaction were not significant (*P* > 0.40). For V*_cmax_*, significant effects were detected for species (χ^2^ = 11.16, df = 3, *P* = 0.011) and the species × day interaction (χ^2^ = 18.16, df = 9, *P* = 0.033). J*_max_* also varied significantly among species (χ^2^ = 32.70, df = 3, *P* < 0.001) and showed a significant species × day interaction (χ^2^ = 23.98, df = 9, *P* = 0.004).

Within-species contrasts to the dry-down endpoint revealed significant post-pulse increases in both *A* and g_sw_ at 4 days after rainfall across all species (*P* < 0.001), while V*_cmax_* and J*_max_* showed no significant changes (Figures 3 and 4). Species comparisons within days indicated that *E. cladocalyx* had higher J*_max_* than other species at 1 and 4 days after rainfall (vs *E. cloeziana*, t = 4.22, *P* < 0.001; vs *E. grandis*, t = 2.69, *P* = 0.048; vs *E. urophylla*, t = 3.48, *P* = 0.006) and higher V*_cmax_* than *E. cloeziana* at both 1 and 4 days after rainfall (t = 2.83, *P* = 0.034; t = 3.20, *P* = 0.013). These results demonstrate that photosynthetic and stomatal parameters tracked the rainfall pulse dynamics, showing significant temporal variation in A and g_sw_ during both cycles, as well as additional species-specific differences in J*_max_* and V*_cmax_*, particularly in *E. cladocalyx* and *E. urophylla* (Figure 4). Generally, *A* and g_sw_ varied temporally and among species, with pronounced differences in V*_cmax_* and J*_max_*. Across both rainfall cycles, V*_cmax_* declined by up to ~ 15% during the post-rain drying, with species-specific changes ranging from small declines to minor increases (−15.4% to +2.0% in December; −14.8% to +0.4% in March). J*_max_* declines were larger in December (−21.6% to −41.6%) but generally smaller or negligible in March (−14.4% to +11.3%). *Eucalyptus cladocalyx* consistently exhibited the highest V*_cmax_* and J*_max_* across all sampling days. Together, these patterns indicate that short-term rainfall pulses produced strong, rapid adjustments in diffusional capacity (stomatal reopening; reflected in *A* and g_sw_), whereas biochemical capacity (V*_cmax_*, J*_max_*) changed more gradually, showing limited sensitivity to short-term soil rewetting. Overall, these results indicate that recovery of leaf water status, stomatal conductance and photosynthetic capacity was temporally decoupled following rainfall, with the magnitude and timing of recovery differing among species and between measurement periods. Mesic species exhibited rapid short-term increases in gas exchange following rainfall, but these increases were not consistently maintained during subsequent dry-down.

### Partitioning of photosynthetic limitation during rainfall pulses

**Figure 5:**
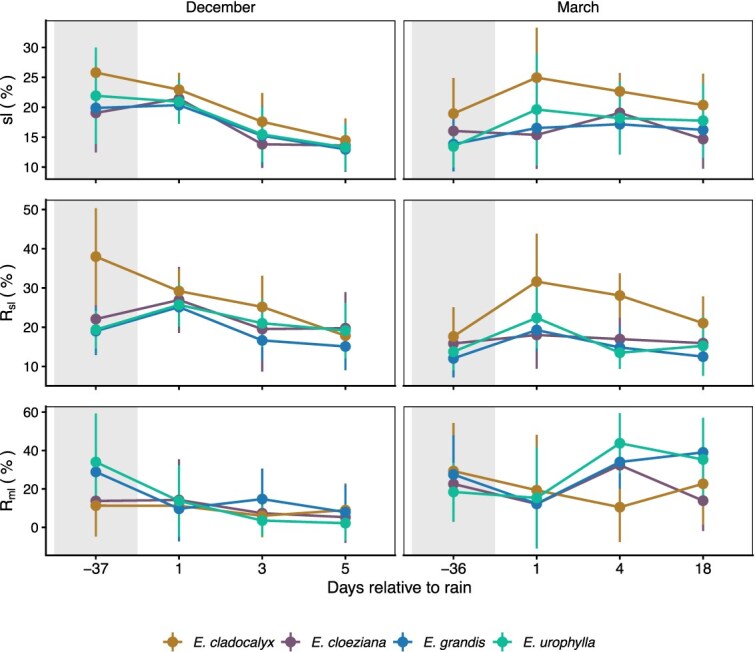
Stomatal limitation (sl), relative stomatal limitation (R_sl_) and relative metabolic limitation (R_ml_) to photosynthesis in four *Eucalyptus* species measured before (shaded) and after (unshaded) rainfall events in December and March. Measurements correspond to 1, 3 and 5 days after rainfall, and 37 days before rain in December, and to 1, 4 and 18 days after rainfall, and 36 days before rain in March (*n* = 5). Shaded regions represent pre-rain (dry-down) conditions, while unshaded regions indicate post-rain recovery. Error bars represent the standard error of the mean.

Consistent with the temporal patterns in photosynthetic and stomatal responses, the R_sl_ and R_ml_ also shifted during the dry-down and rainfall pulse events (Figure 5). In December, R_sl_ varied significantly among species (χ^2^ = 59.09, df = 3, *P* < 0.001) and across measurement days (χ^2^ = 83.38, df = 3, *P* < 0.001), with a significant interaction (χ^2^ = 46.34, df = 9, *P* < 0.001; Figure 5; Table S5 available as Supplementary Data at *Tree Physiology* Online). At the end of the dry-down, *E. cladocalyx* exhibited the highest R_sl_, significantly exceeding *E. grandis* and *E. urophylla* (t = 7.56, *P* < 0.001; t = 7.34, *P* < 0.001). Following the rainfall pulse, R_sl_ declined markedly in *E. cladocalyx* relative to baseline at 1, 3 and 5 days after rainfall (t = 2.91–8.26, Holm *P* ≤ 0.004), whereas *E. grandis* and *E. urophylla* showed transient increases 1 day after rainfall (t = 2.99, *P* = 0.0089; t = 2.91, *P* = 0.0118) that were not maintained thereafter (Figure 5). R_ml_ also varied significantly among species (χ^2^ = 8.79, df = 3, *P* = 0.032) and across days (χ^2^ = 44.00, df = 3, *P* < 0.001), with a significant interaction (χ^2^ = 28.49, df = 9, *P* < 0.001; Table S5 available as Supplementary Data at *Tree Physiology* Online). R_ml_ decreased significantly after rainfall in *E. grandis* (1 day: t = −3.63, *P* = 0.0008; 5 days: t = −3.70, *P* = 0.0008) and *E. urophylla* (1 day: t = −3.31, *P* = 0.0010; 3 days: t < 0.0001; 5 days: t < 0.0001), but remained relatively unchanged in *E. cladocalyx* and *E. cloeziana*. These shifts coincide with the post-rain increases in *A* and g_sw_, indicating that under lower VPD conditions, the December rainfall pulse primarily alleviated stomatal limitation in *E. cladocalyx* and metabolic limitation in *E. grandis* and *E. urophylla*, facilitating recovery of photosynthetic activity.

In March, despite rewetting, high VPD sustained a transient stomatal bottleneck, with R_sl_ again differing significantly among species (χ^2^ = 121.88, df = 3, *P* < 0.001) and across measurement days (χ^2^ = 83.48, df = 3, *P* < 0.001), and a significant interaction (χ^2^ = 25.76, df = 9, *P* = 0.002; Figure 5). R_ml_ followed a similar pattern, showing significant main effects of species (χ^2^ = 23.10, df = 3, *P* < 0.001) and day (χ^2^ = 49.45, df = 3, *P* < 0.001), as well as a significant interaction (χ^2^ = 39.12, df = 9, *P* < 0.001). Relative to the end of the dry-down, R_sl_ increased sharply 1 day after rainfall in *E. cladocalyx*, *E. grandis* and *E. urophylla* (t = 6.63, 4.66 and 4.77; Holm *P* < 0.001), remained elevated at 4 days in *E. cladocalyx* (t = 5.37, *P* < 0.001), and was still higher at 18 days (t = 2.22, *P* = 0.0268; Table S6 available as Supplementary Data at *Tree Physiology* Online). In contrast, R_ml_ declined 1 day after rainfall in *E. cloeziana*, *E. cladocalyx* and *E. grandis* (t = −2.79 to −3.53, Holm *P* ≤ 0.016), indicating rapid relief of biochemical limitation. By 4 days after the pulse, g_sw_ and *A* had increased across species, suggesting delayed stomatal recovery once atmospheric demand moderated. *Eucalyptus urophylla* exhibited elevated R_ml_ later in the cycle (4 days: t = 3.90, *P* = 0.0003; 18 days: t = 2.82, *P* = 0.010), implying a sustained metabolic constraint under persistently high VPD.

Across both measurement cycles, rainfall pulses initially alleviated biochemical constraints first, followed by delayed stomatal recovery under high atmospheric demand. In December, reductions in R_sl_ and R_ml_ corresponded with post-rain increases in A and g_sw_ under low VPD conditions, indicating rapid recovery of photosynthetic capacity when evaporative demand was moderate. In contrast, under higher atmospheric demand, recovery dynamics were temporally decoupled, with biochemical limitation relaxing earlier than stomatal limitation, resulting in delayed increases in gas exchange following rainfall. Together, these patterns demonstrate that rainfall pulses resulted in sequential relief of photosynthetic limitations, with the relative timing and magnitude of recovery governed by atmospheric demand and species-specific water-use strategies. Thus, rainfall pulses first alleviated metabolic limitation before full stomatal reopening, especially under high VPD, revealing a hierarchical recovery pattern. Xeric species showed smaller shifts in both limitation types, indicating coordinated regulation and greater physiological stability during pulse–dry-down cycles.

## Discussion

This study investigated how four *Eucalyptus* species, spanning contrasting climatic origins, regulate and recover photosynthetic function during naturally occurring rainfall pulse–dry-down cycles under field conditions. Specifically, we examined whether species differ in the coordination of plant water status, stomatal and biochemical adjustments following rainfall and subsequent soil drying, and whether recovery dynamics align with expected anisohydric–isohydric strategies. By integrating leaf water potential, gas exchange and photosynthetic limitation partitioning, we identified the sequence and drivers of recovery processes that govern carbon–water coupling during transient rewetting events.

Across both rainfall events, leaf water potential was most negative at the end of the dry-down period and became less negative following rainfall, indicating transient improvement in leaf water status after soil rewetting. However, the magnitude and persistence of this recovery differed among species, consistent with their climatic origins. *Eucalyptus cladocalyx*, native to semi-arid South Australia (Merchant et al. 2007, Johnson et al. 2009), maintained gas exchange at more negative Ψ_leaf_ values than the mesic species, consistent with greater tolerance of hydraulic strain and/or an ability to sustain function at lower water potentials. In contrast, *E. grandis* and *E. cloeziana* maintained less negative Ψ_leaf_, reflecting conservative water-use behaviour that prioritizes hydraulic safety over carbon gain. These contrasting responses fit within the anisohydric–isohydric continuum (Brodribb et al. 2010, Martínez-Vilalta et al. 2014, Meinzer et al. 2017), and align with evidence that xeric *Eucalyptus* species exhibit broader hydraulic operating ranges and larger safety margins than mesic congeners (Héroult et al. 2013, Bourne et al. 2017).

Photosynthetic and stomatal behaviour closely followed these hydraulic adjustments, indicating that rainfall pulses first stimulate rapid diffusional recovery. Across species, both *A* and g_sw_ increased markedly after rainfall, reflecting the reactivation of stomatal function and photosynthetic uptake. As soils re-dried, both parameters declined, with the rate and extent of decline dependent on species and atmospheric demand. *Eucalyptus cladocalyx* maintained higher A and g_sw_ across the full soil-moisture range, whereas *E. grandis* and *E. urophylla* exhibited stronger downregulation as drought intensified, especially under the higher VPD of late summer. These findings confirm that post-rainfall gas-exchange recovery is strongly modulated by atmospheric dryness, which amplifies diffusional limitation even when soils are transiently rewetted (Bourne et al. 2015, Grossiord et al. 2020). Thus, the short-term physiological benefit of rainfall pulses depends not only on soil water replenishment but also on the prevailing evaporative environment. Partitioning of photosynthetic limitation clarified these sequential processes. Stomatal limitation dominated early during drought progression, while metabolic limitation increased as stress intensified. Following rewetting, recovery was hierarchical but strongly modulated by atmospheric demand: under low VPD, stomatal reopening occurred rapidly, whereas under high VPD, biochemical recovery (decline in R_ml_) often preceded full stomatal reopening (decline in R_sl_). Thus, improved leaf water status set the initial boundary conditions for recovery, while the relative timing of diffusional and biochemical reactivation depended on evaporative demand.

Biochemical capacity, represented by V*_cmax_* and J*_max_*, recovered more slowly and less completely than diffusional processes, consistent with evidence that photosynthetic and metabolic recovery can lag behind hydraulic rehydration following drought (Skelton et al. 2017, Ruehr et al. 2019). Increases in J*_max_* and, to a lesser extent, V*_cmax_* following rainfall therefore indicate only partial restoration of electron transport and Rubisco carboxylation capacity. However, the short-lived nature of this enhancement suggests that enzymatic recovery, including the reactivation of Rubisco and repair of the photosynthetic apparatus, is delayed relative to water status and stomatal response (Flexas et al. 2009, Skelton et al. 2017, Ruehr et al. 2019). Such temporal asynchrony has been linked to slow restoration of chloroplast metabolism and the requirement for new protein synthesis after drought stress (Flexas et al. 2004, Morabito et al. 2022). The relative stability of V*_cmax_* in *E. cladocalyx* under declining Ψ_leaf_ further indicates that xeric species maintain enzyme activity and electron-transport efficiency over a broader water potential range, consistent with enhanced biochemical resilience under stress (Cernusak et al. 2011).

Together, these results reveal a continuum of drought-response strategies across the four *Eucalyptus* species, ranging from anisohydric tolerance in *E. cladocalyx* to isohydric regulation in *E. grandis* and *E. urophylla*. *Eucalyptus cladocalyx* maintained photosynthetic function despite low Ψ_leaf_, reflecting a strategy that favours sustained carbon gain under stress, while mesic species achieved high photosynthetic rates under favourable moisture but exhibited pronounced declines during subsequent drying. *Eucalyptus cloeziana* showed limited recovery and low photosynthetic capacity, suggesting reduced flexibility to alternating moisture availability. These interspecific contrasts highlight how species integrate hydraulic safety, stomatal kinetics and biochemical plasticity to cope with pulsed water supply, a key dimension of resilience under increasingly variable rainfall regimes (Allen et al. 2015, Choat et al. 2018).

From a process-based perspective, these findings highlight that rainfall pulses create ephemeral ‘windows of opportunity’ for carbon assimilation that close rapidly as atmospheric demand rises or soils re-dry. The temporal hierarchy of recovery, from hydraulic and diffusional adjustment to delayed biochemical reactivation, explains why post-rain increases in photosynthesis are brief and often decoupled from plant water status. Similar hysteretic coupling between hydraulic recovery and carbon assimilation has been described by Ogle et al. (2015), highlighting the importance of transient physiological feedback during pulse-driven cycles. Such rapid yet transient photosynthetic recovery following rainfall pulses may lead to episodic carbon fluxes that disproportionately contribute to annual productivity, particularly in semi-arid plantations. Incorporating these dynamics into process-based and land-surface models (such as CLM, LPJ-GUESS, ORCHIDEE) will improve predictions of carbon–water coupling, drought legacy effects and the resilience of tree species under increasingly pulsed hydroclimates (Ruehr et al. 2019, Grossiord et al. 2020, Kannenberg et al. 2022). By linking in situ physiological measurements with natural hydrological variability, this study provides a mechanistic foundation for understanding how short-term rainfall events regulate carbon fluxes and recovery dynamics in water-limited forests.

## Conclusion

This study demonstrates that short-term rainfall pulses drive rapid yet transient water potential and photosynthetic recovery in *Eucalyptus* seedlings, with the extent and persistence of recovery governed by both species identity and atmospheric demand. Our results showed that xeric-origin species tolerated greater fluctuations in water potential and sustained photosynthetic activity at lower soil moisture, while mesic species exhibited faster but short-lived recovery following rainfall and sharper declines as the soil dried. These contrasting responses reflect drought strategies along an anisohydric–isohydric continuum (Tardieu and Simonneau 1998, Martínez-Vilalta et al. 2014, Meinzer et al. 2016), confirming that climatic origin shapes the balance between hydraulic safety and carbon gain during pulse–dry-down cycles. The sequence of recovery, hydraulic and stomatal adjustments preceding biochemical reactivation revealed a temporary decoupling between plant water status and photosynthetic metabolism, particularly under high evaporative demand. This asynchrony explains why photosynthetic gains following rewetting are brief and why rainfall pulses contribute disproportionately to short-term carbon fluxes. By integrating natural hydrological variability with mechanistic physiological measurements, this study bridges the gap between controlled drought experiments and field conditions, providing empirical evidence for the hierarchical nature of recovery following natural rainfall events. These insights advance understanding of how rainfall variability influences carbon–water coupling in *Eucalyptus*, highlighting that resilience stems from both the tolerance of water stress and the capacity for rapid, coordinated physiological recovery. Incorporating these recovery kinetics and their species-specific coordination into process-based and land-surface models will improve predictions of forest carbon balance and drought legacy effects under increasingly variable hydroclimates. Integrating such dynamics into mechanistic frameworks will enhance forecasts of drought resilience and carbon fluxes in water-limited forests.

## Supplementary Material

Supplementary_material_EJS_tpag016

## Data Availability

All data supporting the findings of this study are included in the main manuscript and its Supplementary data files. The original data used in this study will be made available from the corresponding author upon request.
