# Supplementary material for: Asynchronous recovery of water relations and photosynthesis following natural rainfall pulses in Eucalyptus
Source: Tree Physiol. 2026 Feb 2;46(3):tpag016. doi: 10.1093/treephys/tpag016 (PMC13016903; doi:10.1093/treephys/tpag016)
Supplement: Supplementary_material_EJS_tpag016 [file supplementary_material_ejs_tpag016.docx]

**Supplementary Table S.1** Estimated marginal means (± SE) of predawn and midday leaf water potential (Ψ_leaf_) for each Eucalyptus species during the December 2024 and March 2025 dry-down cycles. Values were obtained from linear mixed-effects models including species × time as fixed effects and plant ID as a random factor. Degrees of freedom were estimated using the Satterthwaite approximation (‘lmerTest’ R package), and confidence intervals represent 95 % limits on the response scale.

| Species | Month | Time | estimate | SE | df | lower.CL | upper.CL | t-ratio | p-value |
| --- | --- | --- | --- | --- | --- | --- | --- | --- | --- |
| *E. cloeziana* | December | Midday | -1.40 | 0.07 | 124.18 | -1.54 | -1.26 | -20.13 | 6.54E-41 |
| *E. cloeziana* | December | Predawn | -0.86 | 0.08 | 116.16 | -1.02 | -0.69 | -10.44 | 2.14E-18 |
| *E. cladocalyx* | December | Midday | -1.62 | 0.08 | 189.04 | -1.77 | -1.47 | -21.15 | 1.05E-51 |
| *E. cladocalyx* | December | Predawn | -1.07 | 0.06 | 93.85 | -1.20 | -0.95 | -17.18 | 9.89E-31 |
| *E. grandis* | December | Midday | -1.18 | 0.06 | 101.71 | -1.30 | -1.06 | -19.08 | 2.21E-35 |
| *E. grandis* | December | Predawn | -0.56 | 0.12 | 551.69 | -0.78 | -0.33 | -4.85 | 1.62E-06 |
| *E. urophylla* | December | Midday | -1.43 | 0.07 | 141.27 | -1.56 | -1.29 | -20.58 | 2.40E-44 |
| *E. urophylla* | December | Predawn | -0.91 | 0.08 | 111.52 | -1.07 | -0.75 | -11.13 | 8.19E-20 |
| *E. cloeziana* | March | Midday | -1.97 | 0.07 | 134.62 | -2.11 | -1.83 | -28.61 | 4.46E-59 |
| *E. cloeziana* | March | Predawn | -1.40 | 0.08 | 211.01 | -1.55 | -1.24 | -17.88 | 3.93E-44 |
| *E. cladocalyx* | March | Midday | -2.19 | 0.07 | 127.97 | -2.32 | -2.05 | -32.13 | 4.04E-63 |
| *E. cladocalyx* | March | Predawn | -1.60 | 0.08 | 229.53 | -1.76 | -1.44 | -19.98 | 3.93E-52 |
| *E. grandis* | March | Midday | -1.86 | 0.07 | 137.53 | -1.99 | -1.72 | -27.10 | 5.28E-57 |
| *E. grandis* | March | Predawn | -1.38 | 0.08 | 223.46 | -1.53 | -1.22 | -17.30 | 4.15E-43 |
| *E. urophylla* | March | Midday | -1.93 | 0.07 | 134.62 | -2.07 | -1.79 | -28.01 | 5.21E-58 |
| *E. urophylla* | March | Predawn | -1.50 | 0.08 | 229.53 | -1.66 | -1.34 | -18.68 | 5.88E-48 |

**Supplementary Table S2.** Type III Wald χ^2^ tests from mixed-effects models evaluating the effects of **species**, **measurement day**, and their **interaction** on photosynthetic and biochemical traits measured during the December (rainfall-pulse) and March (rewetting) campaigns. Response variables include net photosynthetic rate (**A**), stomatal conductance to water vapour (g_sw_), maximum rate of Rubisco carboxylation (V*_cmax_*) and maximum electron transport rate (J*_max_*). Models were fitted using log-transformed data (with offset adjustments where required) and Kenward–Roger degrees of freedom. Significant main effects (p < 0.05) indicate differences among species or across days, while significant **Species × Day** interactions denote temporal shifts in interspecific responses. Measurements correspond to pre- and post-rainfall days in December and March, reflecting physiological adjustments to soil water availability and atmospheric demand.

| Month | Parameter | Effect | χ^2^ | df | p |
| --- | --- | --- | --- | --- | --- |
| December | A | Species | 10.67 | 3 | 0.0136 |
|  |  | Day | 26.52 | 3 | <0.001 |
|  |  | Species × Day | 12.28 | 9 | 0.198 |
|  | g_sw_ | Species | 12.45 | 3 | 0.006 |
|  |  | Day | 19.01 | 3 | <0.001 |
|  |  | Species × Day | 24.34 | 9 | 0.0038 |
|  | V*_cmax_* | Species | 9.52 | 3 | 0.023 |
|  |  | Day | 7.32 | 3 | 0.062 |
|  |  | Species × Day | 13.59 | 9 | 0.137 |
|  | J*_max_* | Species | 20.98 | 3 | <0.001 |
|  |  | Day | 36.81 | 3 | <0.001 |
|  |  | Species × Day | 21.55 | 9 | 0.01 |
| March | A | Species | 3.19 | 3 | 0.363 |
|  |  | Day | 4081.56 | 3 | <0.001 |
|  |  | Species × Day | 1.99 | 9 | 0.983 |
|  | g*_sw_* | Species | 2.79 | 3 | 0.425 |
|  |  | Day | 53.16 | 3 | <0.001 |
|  |  | Species × Day | 6.83 | 9 | 0.653 |
|  | V*_cmax_* | Species | 11.16 | 3 | 0.011 |
|  |  | Day | 2.1 | 3 | 0.552 |
|  |  | Species × Day | 18.16 | 9 | 0.033 |
|  | J*_max_* | Species | 32.7 | 3 | <0.001 |
|  |  | Day | 0.26 | 3 | 0.967 |
|  |  | Species × Day | 23.98 | 9 | 0.004 |

**Notes:** Mixed-effects models were fitted separately for each month with species and day as fixed effects and tree ID as a random factor.

**Supplementary Table S3**. Pairwise species comparisons within day for net photosynthesis (**A**) and stomatal conductance to water vapour (g_sw_) during the December rainfall-pulse campaign. Measurements were conducted with a LI-6800 across four time points: **37 days before rain**(end-of-dry-down baseline) and **1**, **3**, and **5 days after** rainfall. For each day, Tukey-adjusted pairwise contrasts among species are reported with the back-transformed **estimate**(difference in estimated marginal means), **standard error (SE)**, **degrees of freedom (df)**, **t-ratio**, and **p-value** (Kenward–Roger approximation). The four *Eucalyptus* species are: CX - *E. cladocalyx*, EU - *E. urophylla*, EG - *E. grandis*, and CA - *E. cloeziana*.

| Day | Parameters | Contrast | Estimate | SE | df | t | p |
| --- | --- | --- | --- | --- | --- | --- | --- |
| 37 d before | A | CX − CA | 0.76 | 0.23 | 48.30 | 3.31 | 0.01 |
|  | A | CX − EG | 0.54 | 0.23 | 48.80 | 2.36 | 0.10 |
|  | A | CX − EU | 0.80 | 0.23 | 48.60 | 3.50 | 0.01 |
|  | A | CA − EG | −0.22 | 0.24 | 47.30 | −0.945 | 0.78 |
|  | A | CA − U | 0.03 | 0.23 | 46.90 | 0.13 | 1.00 |
|  | A | EG − EU | 0.25 | 0.23 | 47.70 | 1.09 | 0.69 |
| 1 d after | A | CX − CA | 0.61 | 0.23 | 48.30 | 2.61 | 0.06 |
|  | A | CX − EG | 0.19 | 0.23 | 48.80 | 0.83 | 0.84 |
|  | A | CX − EU | 0.27 | 0.24 | 45.50 | 1.16 | 0.66 |
|  | A | CA − EG | −0.41 | 0.24 | 47.30 | −1.753 | 0.31 |
|  | A | CA − EU | −0.33 | 0.24 | 42.80 | −1.384 | 0.52 |
|  | A | EG − EU | 0.08 | 0.24 | 44.00 | 0.34 | 0.99 |
| 3 d after | A | CX − CA | 0.24 | 0.24 | 45.00 | 1.02 | 0.74 |
|  | A | CX − EG | 0.40 | 0.23 | 49.00 | 1.72 | 0.33 |
|  | A | CX − EU | 0.14 | 0.23 | 48.20 | 0.59 | 0.94 |
|  | A | CA − EG | 0.15 | 0.24 | 45.80 | 0.65 | 0.91 |
|  | A | CA − EU | −0.10 | 0.24 | 43.10 | −0.435 | 0.97 |
|  | A | EG − EU | −0.26 | 0.23 | 48.60 | −1.113 | 0.68 |
| 5 d after | A | CX − CA | 0.23 | 0.23 | 43.30 | 0.98 | 0.76 |
|  | A | CX − EG | 0.38 | 0.23 | 48.80 | 1.70 | 0.33 |
|  | A | CX − EU | 0.12 | 0.23 | 47.30 | 0.55 | 0.95 |
|  | A | CA − EG | 0.15 | 0.24 | 45.80 | 0.65 | 0.91 |
|  | A | CA − EU | −0.10 | 0.24 | 43.10 | −0.435 | 0.97 |
|  | A | EG − EU | −0.26 | 0.23 | 48.60 | −1.113 | 0.68 |
| 37 d before | g_sw_ | CX − CA | 0.98 | 0.23 | 43.20 | 4.30 | 0.00 |
|  | g_sw_ | CX − EG | 0.73 | 0.22 | 45.80 | 3.30 | 0.01 |
|  | g_sw_ | CX − EU | 0.80 | 0.23 | 40.50 | 3.50 | 0.01 |
|  | g_sw_ | CA − EG | −0.25 | 0.23 | 45.80 | −1.096 | 0.69 |
|  | g_sw_ | CA − EU | −0.18 | 0.24 | 41.00 | −0.767 | 0.87 |
|  | g_sw_ | EG − EU | 0.07 | 0.23 | 43.50 | 0.30 | 0.99 |
| 1 d after | g_sw_ | CX − CA | 0.50 | 0.23 | 43.30 | 2.18 | 0.15 |
|  | g_sw_ | CX − EG | −0.03 | 0.22 | 45.90 | −0.132 | 1.00 |
|  | g_sw_ | CX − EU | 0.11 | 0.25 | 37.00 | 0.43 | 0.97 |
|  | g_sw_ | CA − EG | −0.53 | 0.23 | 45.80 | −2.274 | 0.12 |
|  | g_sw_ | CA − EU | −0.39 | 0.26 | 37.60 | −1.496 | 0.45 |
|  | g_sw_ | EG − EU | 0.14 | 0.25 | 39.70 | 0.54 | 0.95 |
| 3 d after | g_sw_ | CX − CA | 0.54 | 0.25 | 37.50 | 2.14 | 0.16 |
|  | g_sw_ | CX − EG | 0.52 | 0.21 | 45.90 | 2.42 | 0.09 |
|  | g_sw_ | CX − EU | 0.46 | 0.23 | 41.60 | 1.99 | 0.21 |
|  | g_sw_ | CA − EG | −0.02 | 0.25 | 38.90 | −0.077 | 1.00 |
|  | g_sw_ | CA − EU | −0.08 | 0.26 | 35.90 | −0.302 | 0.99 |
|  | g_sw_ | EG − EU | −0.06 | 0.23 | 43.30 | −0.265 | 0.99 |
| 5 d after | g_sw_ | CX − CA | 0.53 | 0.25 | 36.70 | 2.11 | 0.17 |
|  | g_sw_ | CX − EG | 0.51 | 0.21 | 45.10 | 2.39 | 0.09 |
|  | g_sw_ | CX − EU | 0.45 | 0.23 | 40.70 | 1.96 | 0.22 |
|  | g_sw_ | CA − EG | −0.02 | 0.25 | 38.90 | −0.077 | 1.00 |
|  | g_sw_ | CA − EU | −0.08 | 0.26 | 35.90 | −0.302 | 0.99 |
|  | g_sw_ | EG − EU | −0.06 | 0.23 | 43.30 | −0.265 | 0.99 |
| 37 d before | V*_cmax_* | CX − CA | 0.71 | 0.19 | 48.50 | 3.69 | 0.00 |
|  | V*_cmax_* | CX − EG | 0.34 | 0.19 | 48.90 | 1.79 | 0.29 |
|  | V*_cmax_* | CX − EU | 0.49 | 0.19 | 48.80 | 2.59 | 0.06 |
|  | V*_cmax_* | CA − EG | −0.37 | 0.20 | 47.50 | −1.880 | 0.25 |
|  | V*_cmax_* | CA − EU | −0.22 | 0.19 | 47.40 | −1.115 | 0.68 |
|  | V*_cmax_* | EG − EU | 0.15 | 0.19 | 48.00 | 0.78 | 0.86 |
| 1 d after | V*_cmax_* | CX − CA | 0.57 | 0.19 | 48.50 | 2.97 | 0.02 |
|  | V*_cmax_* | CX − EG | 0.29 | 0.19 | 48.80 | 1.52 | 0.43 |
|  | V*_cmax_* | CX − EU | 0.32 | 0.19 | 46.30 | 1.65 | 0.36 |
|  | V*_cmax_* | CA − EG | −0.28 | 0.20 | 47.50 | −1.438 | 0.48 |
|  | V*_cmax_* | CA − EU | −0.25 | 0.20 | 43.70 | −1.271 | 0.59 |
|  | V*_cmax_* | EG − EU | 0.03 | 0.20 | 44.70 | 0.15 | 1.00 |
| 3 d after | V*_cmax_* | CX − CA | 0.15 | 0.20 | 45.80 | 0.79 | 0.86 |
|  | V*_cmax_* | CX − EG | 0.31 | 0.19 | 49.00 | 1.63 | 0.37 |
|  | V*_cmax_* | CX − EU | 0.09 | 0.19 | 48.40 | 0.49 | 0.96 |
|  | V*_cmax_* | CA − EG | 0.16 | 0.20 | 46.30 | 0.81 | 0.85 |
|  | V*_cmax_* | CA − EU | −0.06 | 0.20 | 44.10 | −0.308 | 0.99 |
|  | V*_cmax_* | EG − EU | −0.22 | 0.19 | 48.60 | −1.134 | 0.67 |
| 5 d after | V*_cmax_* | CX − CA | −0.01 | 0.19 | 44.00 | −0.054 | 1.00 |
|  | V*_cmax_* | CX − EG | 0.14 | 0.19 | 48.80 | 0.77 | 0.87 |
|  | V*_cmax_* | CX − EU | −0.11 | 0.19 | 47.50 | −0.583 | 0.94 |
|  | V*_cmax_* | CA − EG | 0.15 | 0.20 | 46.30 | 0.79 | 0.86 |
|  | V*_cmax_* | CA − EU | −0.1 | 0.20 | 44.10 | −0.505 | 0.96 |
|  | V*_cmax_* | EG − EU | −0.25 | 0.19 | 48.60 | −1.314 | 0.56 |
| 37 d before | J*_max_* | CX − CA | 0.97 | 0.18 | 47.90 | 5.33 | <0.001 |
|  | J*_max_* | CX − EG | 0.61 | 0.18 | 48.60 | 3.40 | 0.01 |
|  | J*_max_* | CX − EU | 0.73 | 0.18 | 47.90 | 4.08 | 0.00 |
|  | J*_max_* | CA − EG | −0.36 | 0.19 | 47.20 | −1.915 | 0.24 |
|  | J*_max_* | CA − EU | −0.24 | 0.19 | 46.10 | −1.299 | 0.57 |
|  | J*_max_* | EG − EU | 0.12 | 0.18 | 47.20 | 0.63 | 0.92 |
| 1 d after | J*_max_* | CX − CA | 0.77 | 0.18 | 47.90 | 4.22 | 0.00 |
|  | J*_max_* | CX − EG | 0.50 | 0.18 | 48.60 | 2.77 | 0.04 |
|  | J*_max_* | CX − EU | 0.55 | 0.19 | 43.90 | 2.98 | 0.02 |
|  | J*_max_* | CA − EG | −0.27 | 0.19 | 47.20 | −1.438 | 0.48 |
|  | J*_max_* | CA − EU | −0.21 | 0.19 | 41.60 | −1.104 | 0.69 |
|  | J*_max_* | EG − EU | 0.06 | 0.19 | 43.00 | 0.29 | 0.99 |
| 3 d after | J*_max_* | CX − CA | 0.29 | 0.19 | 43.80 | 1.53 | 0.43 |
|  | J*_max_* | CX − EG | 0.39 | 0.18 | 49.00 | 2.16 | 0.15 |
|  | J*_max_* | CX − EU | 0.21 | 0.18 | 47.70 | 1.15 | 0.66 |
|  | J*_max_* | CA − EG | 0.10 | 0.19 | 44.90 | 0.52 | 0.95 |
|  | J*_max_* | CA − EU | −0.08 | 0.19 | 41.50 | −0.407 | 0.98 |
|  | J*_max_* | EG − EU | −0.19 | 0.18 | 48.40 | −0.969 | 0.77 |
| 5 d after | J*_max_* | CX − CA | 0.10 | 0.19 | 42.20 | 0.55 | 0.95 |
|  | J*_max_* | CX − EG | 0.27 | 0.18 | 48.80 | 1.57 | 0.41 |
|  | J*_max_* | CX − EU | −0.004 | 0.18 | 46.80 | −0.024 | 1.00 |
|  | J*_max_* | CA − EG | 0.17 | 0.19 | 44.90 | 0.92 | 0.80 |
|  | J*_max_* | CA − EU | −0.11 | 0.19 | 41.50 | −0.558 | 0.94 |
|  | J*_max_* | EG − EU | −0.28 | 0.18 | 48.40 | −1.535 | 0.43 |

**Supplementary Table S4.** Pairwise species comparisons within day for the maximum rate of Rubisco carboxylation (V*_cmax_*) and maximum electron-transport rate (J*_max_*) during the December rainfall-pulse campaign. Measurements correspond to **37 days before rain** (baseline) and **1**, **3**, and **5 days after** rainfall, representing physiological recovery following rewetting. Tukey-adjusted pairwise contrasts among species are shown as back-transformed **estimates** (differences in estimated marginal means) with **standard error (SE)**, **degrees of freedom (df)**, **t-ratio**, and **p-value**, using Kenward–Roger d.f. The four *Eucalyptus* species are: CX - *E. cladocalyx*, EU - *E. urophylla*, EG - *E. grandis*, and CA - *E. cloeziana*.

| Parameters | Species | 1 d − 37 d | t | p (Holm) | 3 d − 37 d | t | P (Holm) | 5 d − 37 d | t | P (Holm) |
| --- | --- | --- | --- | --- | --- | --- | --- | --- | --- | --- |
| A | CX | 0.29 | 1.58 | 0.38 | −0.039 | −0.19 | 1.00 | −0.05 | −0.27 | 1.00 |
| A | CA | 0.45 | 2.05 | 0.14 | 0.49 | 2.01 | 0.14 | 0.49 | 2.01 | 0.14 |
| A | EG | 0.64 | 2.75 | 0.03 | 0.11 | 0.51 | 1.00 | 0.11 | 0.51 | 1.00 |
| A | EU | 0.81 | 3.41 | 0.00 | 0.62 | 3.29 | 0.01 | 0.62 | 3.29 | 0.01 |
| g_sw_ | CX | −0.09 | −0.84 | 1.00 | −0.08 | −0.66 | 1.00 | −0.09 | −0.79 | 1.00 |
| g_sw_ | CA | 0.40 | 2.50 | 0.06 | 0.36 | 1.38 | 0.35 | 0.36 | 1.38 | 0.35 |
| g_sw_ | EG | 0.67 | 3.63 | 0.00 | 0.13 | 0.87 | 0.78 | 0.13 | 0.87 | 0.78 |
| g_sw_ | EU | 0.60 | 2.31 | 0.08 | 0.26 | 2.27 | 0.08 | 0.26 | 2.27 | 0.08 |
| V*_cmax_* | CX | 0.15 | 0.96 | 0.69 | −0.14 | −0.82 | 0.69 | −0.25 | −1.56 | 0.39 |
| V*_cmax_* | CA | 0.29 | 1.57 | 0.12 | 0.41 | 2.08 | 0.09 | 0.46 | 2.34 | 0.07 |
| V*_cmax_* | EG | 0.20 | 1.04 | 0.91 | −0.11 | −0.63 | 1.00 | −0.06 | −0.33 | 1.00 |
| V*_cmax_* | EU | 0.32 | 1.65 | 0.21 | 0.26 | 1.58 | 0.21 | 0.35 | 2.13 | 0.13 |
| J*_max_* | CX | 0.31 | 2.37 | 0.05 | −0.33 | −2.25 | 0.05 | −0.52 | −3.67 | 0.00 |
| J*_max_* | CA | 0.51 | 3.08 | 0.01 | 0.35 | 1.80 | 0.15 | 0.35 | 1.82 | 0.15 |
| J*_max_* | EG | 0.42 | 2.36 | 0.07 | −0.11 | −0.68 | 0.53 | −0.18 | −1.13 | 0.53 |
| J*_max_* | EU | 0.48 | 2.56 | 0.04 | 0.18 | 1.33 | 0.26 | 0.22 | 1.56 | 0.26 |

**Note:** Estimates for pairwise comparisons are on the back-transformed response scale; test statistics and p-values reflect Kenward–Roger d.f. Tukey adjustment was used for multiple species comparisons within day; Holm adjustment was used for targeted post-rainfall vs baseline contrasts within species.

**Supplementary Table S5.** Type III Wald χ^2^ tests from mixed-effects models assessing the effects of species, measurement day, and their interaction on relative stomatal limitation (R_sl_) and relative metabolic limitation (R_ml_) during the December and March measurement periods. Models were fitted with Kenward–Roger degrees of freedom and logit-transformed, Smithson–Verkuilen–adjusted proportions as the response variable. Significant effects (p < 0.05) indicate differences among species or measurement days, or interactive shifts in limitation partitioning across sampling dates.

| Month | Metric | Term | χ^2^ | Df | p |
| --- | --- | --- | --- | --- | --- |
| December | R_ml_ | Day | 44.004 | 3 | <.001 |
| December | R_ml_ | Species | 8.79 | 3 | 0.03 |
| December | R_ml_ | Species:Day | 28.49 | 9 | <.001 |
| December | R_sl_ | Day | 83.38 | 3 | <.001 |
| December | R_sl_ | Species | 59.09 | 3 | <.001 |
| December | R_sl_ | Species:Day | 46.34 | 9 | <.001 |
| March | R_ml_ | Day | 49.45 | 3 | <.001 |
| March | R_ml_ | Species | 23.10 | 3 | <.001 |
| March | R_ml_ | Species:Day | 39.12 | 9 | <.001 |
| March | R_sl_ | Day | 83.48 | 3 | <.001 |
| March | R_sl_ | Species | 121.88 | 3 | <.001 |
| March | R_sl_ | Species:Day | 25.76 | 9 | <.01 |

**Supplementary Table S6.** Post-hoc pairwise comparisons (Tukey-adjusted) and targeted contrasts (Holm-adjusted) for relative stomatal (R_sl_) and metabolic (R_ml_) limitations across species and measurement days in the December and March measurement periods. Tukey contrasts evaluate species differences within days and day-to-day changes within species; Holm contrasts compare each post-rainfall day to its respective pre-rainfall baseline (37 d before rain in December, 36 d before rain in March). Estimates are presented on the model (logit) scale with Kenward–Roger degrees of freedom. The four *Eucalyptus* species are: CX - *E. cladocalyx*, EU - *E. urophylla*, EG - *E. grandis*, and CA - *E. cloeziana*.

| Month | Metric | Day | Species contrast | Estimate | SE | df | t | p |
| --- | --- | --- | --- | --- | --- | --- | --- | --- |
| December | R_sl_ | 37 days before rain | CA - CX | -0.78 | 0.13 | 314 | 6.05 | <.001 |
| December | R_sl_ | 37 days before rain | CA - EG | 0.18 | 0.13 | 313 | 1.39 | 0.51 |
| December | R_sl_ | 37 days before rain | CA - EU | 0.14 | 0.13 | 314 | 1.10 | 0.69 |
| December | R_sl_ | 37 days before rain | CX - EG | 0.96 | 0.13 | 313 | 7.56 | <.001 |
| December | R_sl_ | 37 days before rain | CX - EU | 0.92 | 0.13 | 313 | 7.34 | <.001 |
| December | R_sl_ | 37 days before rain | EG - EU | -0.04 | 0.13 | 313 | 0.31 | 0.99 |
| December | R_sl_ | 1 day after | CA - CX | -0.14 | 0.13 | 314 | 1.08 | 0.70 |
| December | R_sl_ | 1 day after | CA - EG | 0.06 | 0.13 | 313 | 0.50 | 0.96 |
| December | R_sl_ | 1 day after | CA - EU | 0.04 | 0.13 | 313 | 0.31 | 0.99 |
| December | R_sl_ | 1 day after | CX - EG | 0.20 | 0.13 | 313 | 1.54 | 0.42 |
| December | R_sl_ | 1 day after | CX - EU | 0.18 | 0.13 | 313 | 1.36 | 0.53 |
| December | R_sl_ | 1 day after | EG - EU | -0.02 | 0.13 | 313 | 0.18 | 1.00 |
| December | R_sl_ | 3 days after | CA - CX | -0.38 | 0.12 | 313 | 3.09 | 0.01 |
| December | R_sl_ | 3 days after | CA - EG | 0.13 | 0.13 | 313 | 1.05 | 0.72 |
| December | R_sl_ | 3 days after | CA - EU | -0.16 | 0.13 | 313 | 1.25 | 0.60 |
| December | R_sl_ | 3 days after | CX - EG | 0.51 | 0.13 | 313 | 4.06 | 0.00 |
| December | R_sl_ | 3 days after | CX - EU | 0.22 | 0.13 | 313 | 1.77 | 0.29 |
| December | R_sl_ | 3 days after | EG - EU | -0.29 | 0.13 | 313 | 2.24 | 0.11 |
| December | R_sl_ | 5 days after | CA - CX | 0.06 | 0.12 | 313 | 0.52 | 0.95 |
| December | R_sl_ | 5 days after | CA - EG | 0.29 | 0.13 | 313 | 2.29 | 0.10 |
| December | R_sl_ | 5 days after | CA - EU | 0.00 | 0.12 | 313 | 0.04 | 1.00 |
| December | R_sl_ | 5 days after | CX - EG | 0.22 | 0.12 | 313 | 1.81 | 0.27 |
| December | R_sl_ | 5 days after | CX - EU | -0.07 | 0.12 | 313 | 0.55 | 0.95 |
| December | R_sl_ | 5 days after | EG - EU | -0.29 | 0.13 | 313 | 2.30 | 0.10 |
| December | R_ml_ | 37 days before rain | CA - CX | 0.31 | 0.61 | 313 | 0.50 | 0.96 |
| December | R_ml_ | 37 days before rain | CA - EG | -1.64 | 0.62 | 313 | 2.65 | 0.04 |
| December | R_ml_ | 37 days before rain | CA - EU | -1.82 | 0.61 | 313 | 2.97 | 0.02 |
| December | R_ml_ | 37 days before rain | CX - EG | -1.94 | 0.60 | 313 | 3.22 | 0.01 |
| December | R_ml_ | 37 days before rain | CX - EU | -2.12 | 0.60 | 313 | 3.57 | 0.00 |
| December | R_ml_ | 37 days before rain | EG - EU | -0.18 | 0.60 | 313 | 0.30 | 0.99 |
| December | R_ml_ | 1 day after | CA - CX | 0.02 | 0.61 | 314 | 0.03 | 1.00 |
| December | R_ml_ | 1 day after | CA - EG | 0.28 | 0.60 | 313 | 0.47 | 0.97 |
| December | R_ml_ | 1 day after | CA - EU | -0.12 | 0.60 | 313 | 0.19 | 1.00 |
| December | R_ml_ | 1 day after | CX - EG | 0.26 | 0.62 | 313 | 0.42 | 0.97 |
| December | R_ml_ | 1 day after | CX - EU | -0.13 | 0.62 | 313 | 0.22 | 1.00 |
| December | R_ml_ | 1 day after | EG - EU | -0.40 | 0.61 | 313 | 0.65 | 0.92 |
| December | R_ml_ | 3 days after | CA - CX | 0.25 | 0.58 | 313 | 0.42 | 0.97 |
| December | R_ml_ | 3 days after | CA - EG | -1.12 | 0.60 | 313 | 1.88 | 0.24 |
| December | R_ml_ | 3 days after | CA - EU | 0.70 | 0.60 | 313 | 1.18 | 0.64 |
| December | R_ml_ | 3 days after | CX - EG | -1.37 | 0.60 | 313 | 2.30 | 0.10 |
| December | R_ml_ | 3 days after | CX - EU | 0.46 | 0.60 | 313 | 0.77 | 0.87 |
| December | R_ml_ | 3 days after | EG - EU | 1.83 | 0.61 | 313 | 2.99 | 0.02 |
| December | R_ml_ | 5 days after | CA - CX | -0.83 | 0.58 | 313 | 1.43 | 0.48 |
| December | R_ml_ | 5 days after | CA - EG | -0.72 | 0.60 | 313 | 1.20 | 0.63 |
| December | R_ml_ | 5 days after | CA - EU | 0.53 | 0.59 | 313 | 0.90 | 0.81 |
| December | R_ml_ | 5 days after | CX - EG | 0.11 | 0.59 | 313 | 0.19 | 1.00 |
| December | R_ml_ | 5 days after | CX - EU | 1.36 | 0.58 | 313 | 2.33 | 0.09 |
| December | R_ml_ | 5 days after | EG - EU | 1.25 | 0.60 | 313 | 2.07 | 0.17 |
| March | R_sl_ | 36 days before rain | CA - CX | -0.18 | 0.10 | 395 | 1.81 | 0.27 |
| March | R_sl_ | 36 days before rain | CA - EG | 0.24 | 0.10 | 395 | 2.41 | 0.08 |
| March | R_sl_ | 36 days before rain | CA - EU | 0.08 | 0.10 | 395 | 0.81 | 0.85 |
| March | R_sl_ | 36 days before rain | CX - EG | 0.42 | 0.10 | 395 | 4.23 | 0.00 |
| March | R_sl_ | 36 days before rain | CX - EU | 0.26 | 0.10 | 395 | 2.63 | 0.04 |
| March | R_sl_ | 36 days before rain | EG - EU | -0.16 | 0.10 | 395 | 1.61 | 0.37 |
| March | R_sl_ | 1 day after | CA - CX | -0.76 | 0.14 | 395 | 5.51 | <.0001 |
| March | R_sl_ | 1 day after | CA - EG | -0.12 | 0.14 | 395 | 0.88 | 0.82 |
| March | R_sl_ | 1 day after | CA - EU | -0.29 | 0.14 | 395 | 2.05 | 0.17 |
| March | R_sl_ | 1 day after | CX - EG | 0.63 | 0.14 | 395 | 4.68 | <.0001 |
| March | R_sl_ | 1 day after | CX - EU | 0.47 | 0.13 | 395 | 3.53 | 0.00 |
| March | R_sl_ | 1 day after | EG - EU | -0.16 | 0.14 | 395 | 1.18 | 0.64 |
| March | R_sl_ | 4 days after | CA - CX | -0.65 | 0.14 | 395 | 4.68 | <.0001 |
| March | R_sl_ | 4 days after | CA - EG | 0.17 | 0.14 | 395 | 1.19 | 0.63 |
| March | R_sl_ | 4 days after | CA - EU | 0.27 | 0.14 | 395 | 1.93 | 0.22 |
| March | R_sl_ | 4 days after | CX - EG | 0.82 | 0.14 | 395 | 5.96 | <.0001 |
| March | R_sl_ | 4 days after | CX - EU | 0.92 | 0.14 | 395 | 6.78 | <.0001 |
| March | R_sl_ | 4 days after | EG - EU | 0.10 | 0.14 | 395 | 0.73 | 0.88 |
| March | R_sl_ | 18 days after | CA - CX | -0.43 | 0.13 | 395 | 3.36 | 0.00 |
| March | R_sl_ | 18 days after | CA - EG | 0.16 | 0.14 | 395 | 1.19 | 0.63 |
| March | R_sl_ | 18 days after | CA - EU | 0.02 | 0.13 | 395 | 0.14 | 1.00 |
| March | R_sl_ | 18 days after | CX - EG | 0.59 | 0.13 | 395 | 4.71 | <.0001 |
| March | R_sl_ | 18 days after | CX - EU | 0.45 | 0.12 | 395 | 3.65 | 0.00 |
| March | R_sl_ | 18 days after | EG - EU | -0.14 | 0.13 | 395 | 1.09 | 0.70 |
| March | R_ml_ | 36 days before rain | CA - CX | -0.22 | 0.44 | 395 | 0.50 | 0.96 |
| March | R_ml_ | 36 days before rain | CA - EG | -0.53 | 0.43 | 395 | 1.24 | 0.61 |
| March | R_ml_ | 36 days before rain | CA - EU | 0.32 | 0.43 | 395 | 0.75 | 0.88 |
| March | R_ml_ | 36 days before rain | CX - EG | -0.32 | 0.43 | 395 | 0.73 | 0.89 |
| March | R_ml_ | 36 days before rain | CX - EU | 0.54 | 0.43 | 395 | 1.25 | 0.60 |
| March | R_ml_ | 36 days before rain | EG - EU | 0.86 | 0.43 | 395 | 1.99 | 0.19 |
| March | R_ml_ | 1 day after | CA - CX | -0.29 | 0.60 | 395 | 0.48 | 0.96 |
| March | R_ml_ | 1 day after | CA - EG | -0.18 | 0.62 | 395 | 0.29 | 0.99 |
| March | R_ml_ | 1 day after | CA - EU | -0.35 | 0.61 | 395 | 0.57 | 0.94 |
| March | R_ml_ | 1 day after | CX - EG | 0.11 | 0.59 | 395 | 0.19 | 1.00 |
| March | R_ml_ | 1 day after | CX - EU | -0.05 | 0.59 | 395 | 0.09 | 1.00 |
| March | R_ml_ | 1 day after | EG - EU | -0.17 | 0.60 | 395 | 0.28 | 0.99 |
| March | R_ml_ | 4 days after | CA - CX | 2.30 | 0.61 | 395 | 3.77 | 0.00 |
| March | R_ml_ | 4 days after | CA - EG | -0.44 | 0.62 | 395 | 0.71 | 0.89 |
| March | R_ml_ | 4 days after | CA - EU | -0.89 | 0.61 | 395 | 1.46 | 0.46 |
| March | R_ml_ | 4 days after | CX - EG | -2.73 | 0.60 | 395 | 4.55 | <.0001 |
| March | R_ml_ | 4 days after | CX - EU | -3.19 | 0.59 | 395 | 5.38 | <.0001 |
| March | R_ml_ | 4 days after | EG - EU | -0.45 | 0.60 | 395 | 0.76 | 0.87 |
| March | R_ml_ | 18 days after | CA - CX | -0.56 | 0.56 | 395 | 1.01 | 0.75 |
| March | R_ml_ | 18 days after | CA - EG | -2.15 | 0.60 | 395 | 3.57 | 0.00 |
| March | R_ml_ | 18 days after | CA - EU | -1.78 | 0.59 | 395 | 3.03 | 0.01 |
| March | R_ml_ | 18 days after | CX - EG | -1.58 | 0.55 | 395 | 2.87 | 0.02 |
| March | R_ml_ | 18 days after | CX - EU | -1.22 | 0.54 | 395 | 2.26 | 0.11 |
| March | R_ml_ | 18 days after | EG - EU | 0.37 | 0.58 | 395 | 0.63 | 0.92 |
